# Physico-chemical characteristics of gamma-irradiated gelatin

**DOI:** 10.1007/s40204-014-0021-z

**Published:** 2014-03-07

**Authors:** Md. Minhajul Islam, Asaduz Zaman, Md. Shahidul Islam, Mubarak A. Khan, Mohammed Mizanur Rahman

**Affiliations:** 1 Department of Applied Chemistry and Chemical Engineering, Faculty of Engineering and Technology, University of Dhaka, Dhaka, 1000 Bangladesh; 2 Institute of Radiation and Polymer Technology (IRPT), Atomic Energy Research Establishment (AERE), P. O. Box No. 3787, Dhaka, 1000 Bangladesh

**Keywords:** Solid gelatin, Gamma irradiation, Crosslinking, Radiation degradation, Biomedical application

## Abstract

This article reports the effects of gamma irradiation (dose ranges 0.1–10 kGy from ^60^Co source) on the characteristics of solid gelatin and the physico-mechanical, microstructural and bioactive properties of the scaffold prepared from irradiated gelatin solution. FTIR, intrinsic viscosity, bloom strength, thermal properties, SEM, tensile properties, water uptake ability and antimicrobial activities of non-irradiated and irradiated solid gelatin and its scaffolds were investigated. The detailed experimental results for the solid gelatin demonstrated that 1 kGy γ-irradiated samples showed higher intrinsic viscosity, enhanced thermal stability and bloom strength than other irradiated samples. Furthermore, the scaffold thus prepared from irradiated and non-irradiated gelatin also revealed that 1 kGy samples showed the highest tensile strength and modulus with good water resistivity than other irradiated and non-irradiated samples. In addition to the physico-mechanical properties, 1 kGy scaffolds have also exhibited the highest resistivity towards microbial growth that can have potentiality as scaffold in biomedical sector. The enhanced functional and bioactive properties at low irradiation doses (1 kGy) may occurred due to an initial breaking of hydrogen bonds of polypeptide chains in gelatin molecules that indicated by the shift of amide A, I and II peaks to higher wave numbers in FTIR. This enhancement resulted probably due to the domination of crosslinking over degradation at 1 kGy. It was also observed that 1 kGy γ-radiation-induced crosslinking has lowered the hydrophilicity by decreasing water uptake and mean pore diameter of the interconnected porous structures of gelatin.

## Introduction

Gelatin is a naturally occurring macromolecular and biodegradable protein material that is produced by the controlled partial hydrolysis of collagen synthesized from skins, white connective tissues and bones of animals which is composed of amino acid residues at different proportions and combinations (Marfil et al. 2012). As a biocompatible, biodegradable, and edible material, gelatin has been used as starting materials in a variety of applications through its biosafety verified in foods and clinical fields for a long time (Inamura et al. 2010; Sultana et al. 2010). Because of the favorable properties, such as high water solubility, non-toxicity, thermo-reversible sol–gel transition, high mechanical strength and elasticity in a dry state, moisturizing cause by binding a plenty of water molecules and admixture of small particles in water, gelatin are widely used in foods, water-soluble capsules, coating materials for oral drugs, stabilizer of photo-sensitive reagents in photographic films, adsorbent for diluted chemicals and adhesive agents (Bessho et al. 2005). Furthermore, through many functional side groups of amino acids, gelatin readily undergoes chemical crosslinking, that made it suitable for its application as a biomaterial, and these advantages made gelatin-based controlled release systems in diverse fields ranging from tissue engineering to drug delivery and gene therapy (Rahman et al. 2013).

Gelatin as a hydrophilic biopolymer specifically interacts with water and undergoes drastic changes of its physico-mechanical properties depending on the moisture content (Kozlov and Burdygina 1983), i.e., its poor mechanical properties, especially when exposing to wet and humid conditions limit its application in many areas (Zhang et al. 2010). Therefore, gelatin materials for long-term biomedical applications must be submitted to crosslinking using a wide variety of chemical and physical crosslinking techniques, that improves both the thermal and the mechanical stability as well as water-resistant properties of this biopolymer (Bigi et al. 2004). Physical crosslinking methods include dehydrothermal treatment, ultraviolet, and γ-irradiation (Jo et al. 2005). Of these methods, γ-irradiation has become well known as a very convenient tool for the improvement of the mechanical properties, chemical resistance, thermal stability, melt flow and other important properties of polymer materials through a crosslinking, grafting and degradation techniques (Inamura et al. 2010; Piermaria et al. 2008).

Gamma irradiation has long been employed for decontamination and/or sterilization of dehydrated vegetables, fruits (Fu et al. 2000), seasonings (Dorozhkin 2009), and animal foods (Yang et al. 1993) and then to prolong the storage period of the irradiated food. Many countries in the world are engaged in the commercialization of irradiation food according to the Bulletin of the Meeting of Combined Expert Committee of FAO, IAEA and WHO. The merit of radiation-induced crosslinking by γ-rays is that no residual chemical reagent, such as toxic crosslinkers, remains in the irradiated gelatin that can be used in biomedical and food applications. Irradiation caused irreversible changes at the molecular level by breakage of the covalent bonds of the polypeptide chains of gelatin at solution stage. The protein fragmentation in aqueous solutions is affected by the local conformation of an amino acid in the protein, its accessibility to the water radiolysis products, and the primary amino acid sequence (Filali-Mouhim et al. 1997). The application of solid gelatin in food and pharmaceuticals is extensive; however, the effect of the γ-radiation on the variation of different functional and bioactive properties of solid gelatin has not investigated. Furthermore, another novelty of this work is that it demonstrated effect of γ-radiation on the physico-mechanical and bioactive properties of the scaffold film produced from irradiated gelatin to that of the scaffold prepared from non-irradiated gelatin.

## Experimental

### Materials

Gelatin granules (type B from cattle bones, Bloom strength-240 g, and pharmaceutical grade) were supplied by the Global Capsules Limited, Barishal, Bangladesh and Co-60 gamma sources were used to generate γ-irradiation at the Atomic Energy Research Establishment (AERE), Savar, Dhaka, Bangladesh. Solid gelatin granules were irradiated with different doses (0.1–10 kGy) at a dose rate of 5.5 kGy/h.

### Methods

#### Characterization of the irradiated solid gelatin samples

FTIR spectra of the dried scaffolds were recorded using a spectrophotometer (Perkin-Elmer RX 1, England) within the spectral region of 400–4,000 cm^−1^. Solutions of different concentrations 0.2–1 % w/v, respectively, of non-irradiated and irradiated gelatin were prepared in de-ionized water at 30 °C temperature and maintained at that temperature in a water bath. The relative viscosity, *η*_rel_ of the solutions was determined at 30 °C with an automated solution viscometer (Ostwald viscometer) by measuring flow times of the pure solvent and polymer solutions. In brief, approximately 5 cm^3^ of the liquid was injected into the viscometer and equilibrated to a constant temperature maintained within an accuracy of ±0.5 °C for about 10 min. Then, the flow times of the pure solvent (*t*_2_) and polymer solutions (*t*_1_) were measured and using these data, the reduced viscosities, *η*_red_ = (*η*_rel_−1)/c of these solutions were calculated; where c is the concentration. Hence, from the plots of *η*_red_ versus the concentration, we have determined the intrinsic viscosity [η] of the gelatin samples.

The bloom strengths of the gelatin samples were determined according to British Standard BS 757 (Bhat and Karim 2009). Gelatin was weighed into a bloom jar and dissolved in distilled water to have concentration of 6.67 % (w/v). After inserting a perforated stopper, the sample was allowed to hydrate for 1–3 h at room temperature. Then the sample jar was placed in the 65 °C bath and stirred periodically for approximately 15 min to dissolve gelatin completely. Later it was left to condition for 16 h in a water bath at 10 °C. After conditioning, the bloom jar was centrally placed under a 1.3 cm diameter flat bottomed AOAC plunger attached to a Stevens LFRA Texture Analyzer. The maximum force (g) taken by the plunger with 5 kg load cell to penetrate 4 mm onto gelatin gel’s surface at a crosshead speed of 1 mm/s was recorded as the bloom strength. Values were averaged including standard deviation of at least five specimens.

Thermal degradation measurements of both γ-ray-irradiated and non-irradiated gelatin were carried out using a TG/DTA 6300 system controlled to an EXSTAR 6000 STATION, Seiko Instrument, Inc. Japan. Samples of about 2.5 mg kept into aluminum cell were heated in the temperature range of 30–600 °C at a heating rate of 20 °C/min under nitrogen atmosphere. Different phase transition temperatures as well as the amount of enthalpy associated with them were measured using a DSC-60, Shimadzu Corp., Japan. All samples (both γ-ray-irradiated and non-irradiated gelatin) of approximately 2.5 mg were sealed in an aluminum pan. Then heating was carried out at a rate of 10 °C/min within the temperature range of 30–500 °C and during the measurement, ultra-pure nitrogen was used as a purge gas at a flow rate of 20 cm^3^min^−1^.

#### Preparation of gelatin scaffolds

Both non-irradiated and γ-ray-irradiated solid gelatin granules (10 g) were dissolved in 100 cm^3^ of de-ionized water with constant stirring and heating at 50 °C temperature for 45 min to prepare 10 % (w/v) gelatin solutions. Then equal amount (5 cm^3^) of each warm solution was poured on to polystyrene sheet made frames (20 × 10 × 1.5 cm^3^) to prepare gelatin scaffolds. The scaffolds were allowed to dry under laminar air flow at room temperature in a sterile condition for 24 h. The dried, smooth and transparent scaffolds of about 0.3 mm thickness were peeled off and then preserved in the desiccators for tensile and water uptake tests.

#### Characterization of the gelatin scaffolds

##### Mechanical properties

The tensile properties such as tensile strength (TS), tensile modulus (TM) and elongation at break (Eb %) of the scaffolds of γ-ray-irradiated and non-irradiated gelatin were measured using a universal testing machine (model: H50KS-0404, Hounsfield Series S, UK) with 5,000 N load range at a crosshead speed of 20 mm/min. The specimens were cut into dog-bone shape and then conditioned at 25 °C and 65 % relative humidity for 10 days before performing the test. Specimens, type IV (ASTM-D638) had a rectangular cross section of 6 mm and a gage length of 40 mm with a thickness of 0.3 mm.

##### Water uptake

To measure the equilibrium water uptake ability of both γ-ray-irradiated and non-irradiated gelatin scaffolds, previously weighed samples were immersed into de-ionized water at room temperature. Swollen samples were then taken out carefully at regular intervals (4, 8, 12, 16 and 20 min); the surface water was removed by filter paper and weighed until constant weight was obtained. Thus, the experiment was carried out until equilibrium was attained and the water uptake ability was determined according to the equation: water uptake (%) = (*W*_WET_ − *W*_DRY_)/*W*_DRY_ × 100 %; where, *W*_WET_ = weight of film after water uptake and *W*_DRY_ = initial dry weight of the film.

##### Surface morphology

For morphological study, 18 %(w/v) solutions of γ-ray-irradiated (0.5, 1 and 2.5 kGy) and non-irradiated gelatin granules were prepared at 50 °C and then the solutions were refrigerated at −80 °C for 3 h and freeze dried at −50 °C for 36 h. The dried scaffolds were platinum coated (to make them conductive) using a JEOL JFC-1,600 auto fine coater and the internal structures were inspected at an accelerating voltage of 10 kV using a JEOL JSM-6490 LA analytical scanning electron microscope (both instruments from JEOL, Japan). The pore length and width were measured by a stereomicroscope (Euromax, Gemany) equipped with an optical micrometer. The sizes of the pores were calculated using the expression *d* = √(*l* × *h*), where *l* and *h* are the average length and width of the pores, respectively. At least 40 pores were assessed and the values were statistically analyzed and expressed as the mean ± standard deviation according to the method described in the literature (Kang et al. 1999; Velema and Kaplan 2006).

##### Antimicrobial activity

Antimicrobial activity of irradiated and non-irradiated gelatin was investigated against *Bacillus cereus* and *Escherichia coli* by the disc diffusion method. This method was done in Muller Hinton medium. The media used for antimicrobial activity was poured into sterile petri plate and was allowed to be cooled. Then the test cultures (*Bacillus cereus* and *Escherichia coli*) were inoculated properly onto the media. Gelatin samples were autoclaved for 2 h to remove any bacterial contamination. Besides, 1 g of solid gelatin granules (either γ-irradiated or non-irradiated) was dissolved into 9 cm^3^ normal saline and from each solution, 0.1 cm^3^ sample was placed onto the holes (6 mm diameter) made on the *B. cereus* and *E. coli* cultured agar plate. Subsequently, these plates were incubated overnight at 37 °C and the inhibition zone was measured for the evaluation of antimicrobial activity.

### Statistical analysis

Five replicates were carried out in each experiment of mechanical properties. All data were analyzed by SPSS software, version 15 using one-way ANOVA analysis. The level of statistical significance was set at 5 % (*p* < 0.05).

## Results and discussion

### Characterization of solid gelatin

#### FTIR analysis

The chemical shift of amide A, I and II for non-irradiated and γ-irradiated chitosan is given in Table 1. With the increase of γ-irradiation dose, the absorption for hydrogen bonded N–H at 3,327 cm^−1^ for non-irradiated gelatin was shifted to lower wave number to 3,319 and 3,335 cm^−1^ for 0.5 and 1 kGy doses, respectively. The results also showed that the absorption band at 1,656 cm^−1^ of C=O stretching (amide I) for non-irradiated gelatin has also shifted to 1,644 cm^−1^ (broad) for 0.5 kGy dose and then to higher wave number at 1,657 cm^−1^ (broader) for 1 kGy dose. Furthermore, the absorption for N–H bending (amide II) coupled with C–N stretching showed the similar trend to that of amide I throughout the studied irradiation ranges. It was also revealed from the Table 1 that due to the increase of radiation doses more than 1 kGy, amide A, I and II peaks have shifted to the lower wave number gradually due to the degradation of protein molecules. The shift of absorption of amide A, I and II peaks for 1 kGy radiation doses with increased intensity is associated with crosslinking and due to the formation of hydrogen bond between the amino acid residues of the chains.

**Table 1:** Variation of peak wave numbers (cm^−1^) of amide A, I and II functional groups of gelatin with respect to absorbed dose

γ-irradiation doses(kGy)	N–H stretch, (amide A) coupled with H bonding	C=O stretch (amide I)	N–H bending (amide II) coupled with C–N
0	3,327	1,656	1,558
0.5	3,319 (broad)	1,644 (broad)	1,557
1	3,335 (broader)	1,657 (broader)	1,558 (broad)
2.5	3,327	1,655	1,558
5	3,326	1,654	1,557
10	3,326	1,653	1,552

The initial changes for 0.5 kGy are caused by an initial breaking of hydrogen bonds of the gelatin molecules due to denaturation or degradation phenomena. However, for further increase in irradiation doses, the crosslinking phenomenon started to dominate over degradation/denaturation process.

#### Intrinsic viscosities

**Fig. 1:**
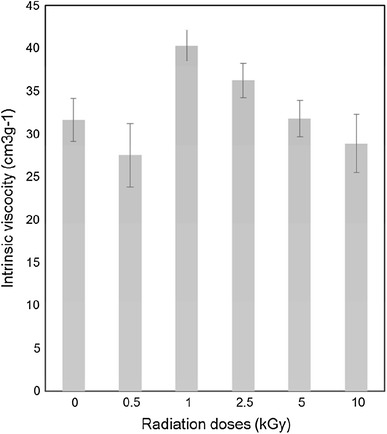
Variation of intrinsic viscosity of gelatin with different doses of γ-irradiation

The average values of intrinsic viscosities for non-irradiated and γ-irradiated gelatin samples are graphically presented in Fig. 1. It was observed that by applying 0.5 kGy γ-radiation, the intrinsic viscosity has decreased from 30.9 cm^3^/g for non-irradiated gelatin to 26.8 cm^3 ^g^−1^ which happened probably due to the rupture of the peptide linkages (degradation) of the gelatin by irradiation. However, the viscosity then increased to 40.1 cm^3 ^g^−1^ for 1 kGy irradiated samples and then started to decrease again with increasing irradiation doses to 10 kGy. The lowest average viscosity value of 28.8 cm^3 ^g^−1^ that is even lower than non-irradiated gelatin was obtained for 10 kGy samples.

This above result also supports the phenomena that at lower radiation dose (<1.0 kGy) the denaturation/degradation is dominated over crosslinking and irradiation dose of 1 kGy crosslinking is dominated over degradation. The change of intrinsic viscosities due to the effect of γ-irradiation may be explained through a combination of moderate degradation and chain scission and that was responsible for this lowering of viscosity at high radiation doses.

#### Bloom strength

**Fig. 2:**
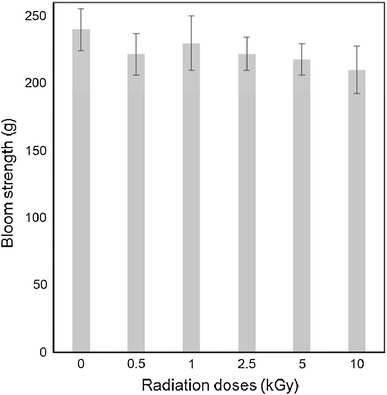
Variation of bloom strength of gelatin with different γ-irradiation doses

The bloom strength for all γ-ray-irradiated gelatin (as shown in Fig. 2) samples were found lower than its original value 240 g for non-irradiated gelatin. The results also showed that there are significant decrease of bloom strength (221 g) for 0.5 kGy gelatin, however, it increased to 230 g for 1 kGy dose and again decreased to 210 g for 10 kGy dose. This result also supports the findings obtained in the previous sections that the crosslinking phenomena was dominated over degradation for enhanced characteristics given by 1 kGy samples than other irradiated samples.

#### Thermal analysis

The denaturation temperature, glass transition temperature, crystallization temperature, melting temperature, decomposition temperature and associated enthalpy changes for γ-ray-irradiated and non-irradiated gelatin are given in Table 2. The first endothermic phase transition of non-irradiated gelatin took place at 101 °C that was assigned as the denaturation of gelatin. The denaturation temperatures of γ-irradiated gelatin were found from 91 °C (for 0.2 kGy dose) which then increases as the radiation doses are increased and the detection of this transition is not easy with a hydrophilic material as water is also lost in the same temperature range (Rahman et al. 2013). The second endothermic phase transition of non-irradiated gelatin took place near 155 °C that was assigned to the glass transition followed by a slight indication for exothermic crystallization at 201 °C. The next endothermic phase transition of non-irradiated gelatin took place near 229 °C and is the low temperature melting (Mukherjee and Rosolen 2013) followed by another high temperature melting at 262 °C. The final endothermic phase transition of non-irradiated gelatin took place near 327 °C that should be assigned to the decomposition or cracking of gelatin.

**Table 2:** Transition temperatures and associated enthalpies of both γ-ray-irradiated and non-irradiated gelatin by DSC

γ- doses (kGy)	Denaturation		Glass-phase transition	Crystallization	Melting-phase transitions		Decomposition or cracking	
	*T*_D_ (°C)	∆*H*_D_ (J/g)	*T*_g_ (°C)	*T*_C_ (°C)	*T*_ML_ (°C)	*T*_MH_ (°C)	*T*_d_ (°C)	∆*H*_d_(J/g)
0	101	54	155	201	229	262	327	161
0.5	98	85	188	210	238	265	333	197
1	103	85	199	222	243	280	343	203
2.5	98	84	203	208	220	270	338	215
10	92	51	177	203	214	265	327	184

*T*_D_ denaturation temperature, *T*g glass transition temperature, *T*_C_ crystallization temperature, *T*_ML_ low melting temperature, *T*_MH_ High melting temperature, *T*_d_ decomposition temperature, ∆*H* enthalpies associated with phase transition

**Fig. 3:**
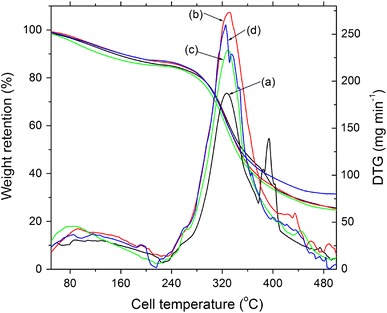
TGA and DTG thermogram of **a** non-irradiated, **b** 1 kGy, **c** 2.5 kGy and **d** 10 kGy γ-ray-irradiated gelatin sample

The denaturation temperature of gelatin slightly decreased to 98 °C at 0.5 kGy than non-irradiated (101 °C) through helix to coil transformation; but with the increase of irradiation doses, it reaches 103 °C at 1.0 kGy doses due to crosslinking. After initial slight increase of melting and decomposition temperature for 0.5 kGy through chain scission, a significant rise of these transition temperatures was observed at 1 kGy. Generally, the degree of crosslinking increases the thermal stability of gelatin, as shown by the shift of the melting and denaturation temperature to higher values up to1 kGy γ-ray irradiation. This result resembles well enough with the variation of enthalpies associated with the melting and denaturation with γ-irradiation dose (Dorozhkin 2009). TGA thermograms of γ-ray-irradiated and non-irradiated gelatin are presented in Fig. 3. The first stage, continuing up to about 120 °C, was related to the evaporation of the absorbed and bound water as well as NH_3_ and CO_2_ gases from the gelatin (Rahman et al. 2013).

The difference in the thermal decomposition behavior of the gelatin may be interpreted more clearly from the DTG curves as shown in Fig. 3. The DTG curves showed two peak temperatures corresponding to first and second stage of mass loss. The maximum rate of decomposition of non-irradiated gelatin was found at 327 °C, whereas for 1 kGy gelatin it attained the highest value at 342 °C. From the TGA curves, the least weight loss of 72.4 % was obtained for 10 kGy sample, however, non-irradiated has a weight loss of 78.4 %.

### Characterization of gelatin scaffold

#### Tensile properties of the gelatin scaffolds

**Fig. 4:**
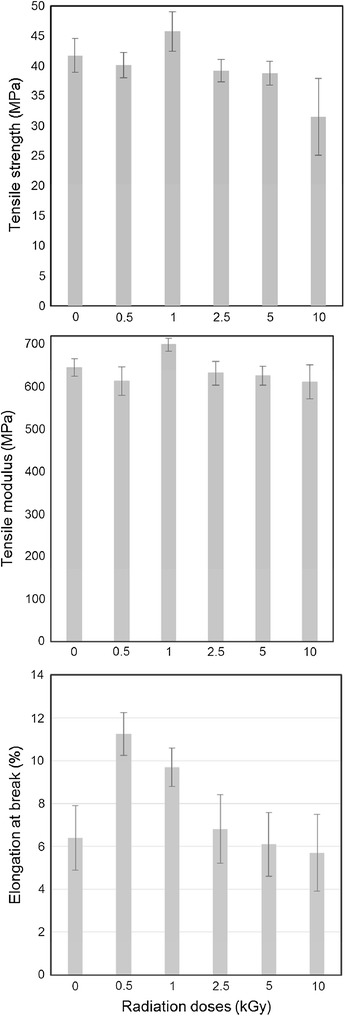
Variation of tensile strength, elongation at break and tensile modulus gelatin’s film at various γ-irradiation doses

The variations of tensile properties of gelatin scaffold prepared from γ-irradiated gelatin and non-irradiated gelatin samples are graphically presented in Fig. 4. The *T*_S_ of the non-irradiated gelatin scaffold was 41.8 MPa, however, it decreases slightly for 0.5 kGy and then increases for 1 kGy samples. After gaining the highest value 44.7 MPa for 1 kGy irradiation the *T*_S_ value then decreases with increasing irradiation doses. The lowest *T*_S_ (30.8 MPa) was observed for the scaffold prepared from 10 kGy irradiated gelatin. Similar to the *T*_S_ value, the highest *T*_M_ 700 MPa was obtained for 1 kGy γ-ray-irradiated gelatin scaffold. Other results of *T*_M_ followed same trend to that of *T*_S_. Generally, the higher the *T*_S_, the lower the elongation at break is observed. The elongation at break for non-irradiated gelatin was found to be 6.2 %, however, by applying 0.5 kGy gamma-irradiation dose, elongation at break values significantly increased to 11.4 % and is the highest among all irradiated samples. The lowest elongation of 5.8 % among the irradiated samples was given by 10 kGy samples.

**Fig. 5:**
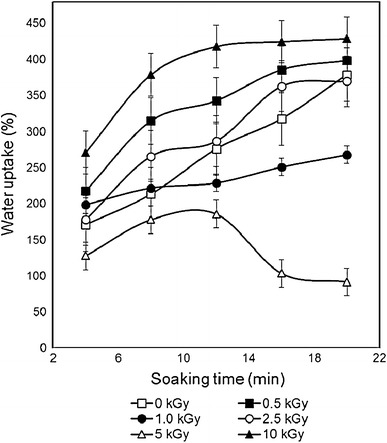
Variation of water uptake of gelatin with different doses of γ-irradiation

#### Water uptake

As gelatin is a hydrophilic material, water uptake was quite rapid at first few minutes and then it became slow or either attained almost plateau, but after 20 min of soaking and at this relatively large soaking in water, gelatin scaffolds became almost saturated in water and began to form gel. The water uptakes of both non-irradiated and γ-irradiated gelatin at different soaking times are represented in Fig. 5. It was observed that there is no particular sequence for water absorption behavior and 2.5 kGy samples showed the lowest water uptake followed by 1 kGy irradiated sample.

**Fig. 6:**
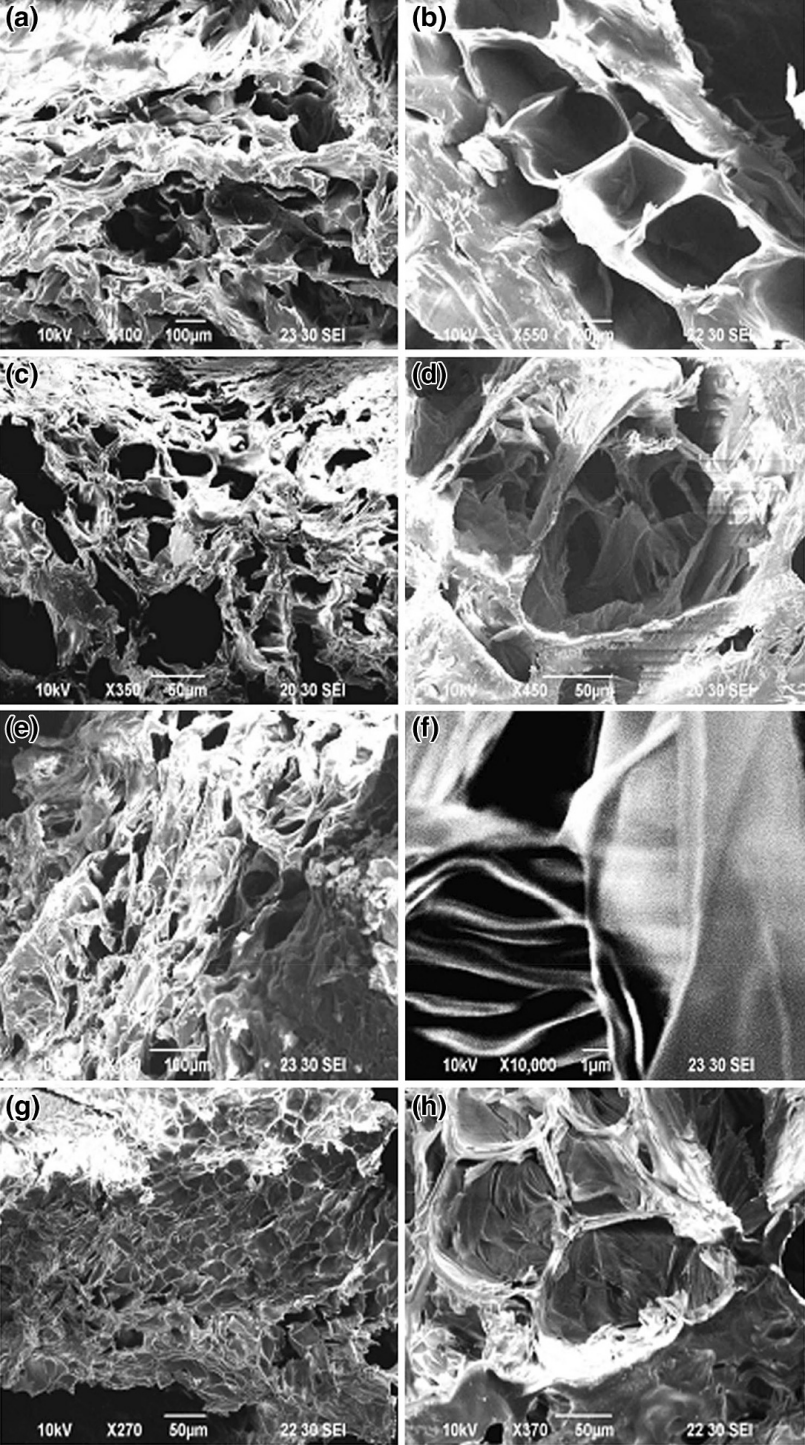
SEM micrographs of freeze-dried scaffolds of **a**, **b** non-irradiated; **c**, **d** 0.5 kGy; **e**, **f** 1 kGy; and **g**, **h** 10 kGy γ-ray-irradiated gelatin

The water uptake of 0.5 kGy γ-ray-irradiated gelatin was found higher than that of non-irradiated gelatin and then further increment of γ-irradiation dose produced lower water uptake for 1 and 2.5 kGy γ-ray-irradiated gelatin, respectively. This suggests that the higher radiation dose up to 2.5 kGy has induced crosslinking reaction along with lowering of the void space within the gelatin structure that hindered the water to get into the scaffolds that is in good agreement with the SEM results.

#### Microstructural properties

The SEM images (as represented in Fig. 6) were used to characterize the microstructure of non-irradiated and irradiated (0.5, 1 and 2.5 kGy) and freeze-dried gelatin solutions. The SEM images suggest that the gelatin is porous, with three-dimensional interconnected microstructures resembling other natural macromolecular scaffold structures. The interconnection of the pores of gelatin scaffold may be assigned to the three-dimensional network formation due to crosslinking. The scaffolds of 0.5 and 1 kGy samples have smaller (mean pore diameter: 60 ± 13 and 50 ± 9 μm, respectively) and interconnected pores in their microstructure than that (mean pore diameter: 87 ± 11 μm) of the non-irradiated samples. The amide bonding within the gelatin molecules determines its pore size that may decrease through increase in crosslinking. Hence, for the 1 kGy γ-ray-irradiated gelatin, smaller size of the pores could be assigned to the more crosslinking of polypeptide chains and may be advantageous for the preparation of artificial bone.

#### Antimicrobial properties

Antibacterial activity of the scaffold prepared from gelatin irradiated at different γ-radiation doses was evaluated and compared with the controlled sample (non-irradiated gelatin). The γ-irradiated gelatin inhibits the growth of *B. cereus* and *E. coli* effectively and the necessary dose could be decreased up to 1.0 kGy. The giant cells of *B. cereus* and *E. coli* observed by the treatment of irradiated gelatin suggested that the irradiated gelatin attached on the cell surface and inhibited the cell division. Anti-fungal activity was also induced but this was lower than the antibacterial activity. The results of inhibition zone of *E. coli* for non-irradiated and irradiated gelatin are given in Table 3.

**Table 3:** Inhibition zone of *E. coli* for non-irradiated and irradiated (at different gamma doses) gelatin

Samples (kGy)	Inhibition zone (mm)	Standard deviation
0	11	±0.54
0.5	7	±1.04
1	6	±0.71
2.5	6	±1.24
5	7	±1.10
10	8	±0.94

## Conclusion

The above research can be concluded into the following points:

1. Solid gelatin was γ-irradiated at different doses (0.5–10 kGy) and its various characteristics were measured and compared with non-irradiated samples. It was observed that 1 kGy irradiated gelatin yielded better characteristics such as viscosity, bloom strength and thermal stability than other irradiated samples.
2. Scaffold film of irradiated and non-irradiated gelatin was prepared from water solution and its tensile properties, water resistivity, microstructural and bioactive properties were described with respect to irradiation doses. The results demonstrated that significant improvement of tensile properties and antimicrobial properties were obtained from 1 kGy irradiated sample than others.

This enhanced functional and bioactive properties at low irradiation doses that may have occurred due to an initial breaking of hydrogen bonds of polypeptide chains of gelatin molecules (indicated by the shift of amide A, I and II absorption band to lower wave numbers in FTIR) in which crosslinking phenomenon started to dominate over degradation process (indicated by the shift of amide A, I and II peaks to higher wave numbers in FTIR) and this domination is the most effective at 1 kGy irradiation dose.
